# On a Case of Nervous Affection Cured by Pressure of the Carotids; with Some Physiological Remarks

**Published:** 1811-12

**Authors:** C. H. Parry


### *On a Case of nervous Affection cured by Pressure of the Carotids; with some physiological Remarks.* By C. H. Parry, *M. D. F. R. S.*

(Phil. Trans. P. 1. 1811.)

About the year 1786, I began to attend a young lady, who laboured under repeated and violent attacks, either of head-ach, vertigo, mania, dyspnœa, convulsions, or other symptoms usually denominated nervous. This case I described at large to the Medical Society of London, who published it in their Memoirs, in the year 1788. Long meditation on the circumstances of the case, led me to conclude, that all the symptoms arose from a violent impulse of blood into the vessels of the brain; whence I inferred, that as the chief canals conveying this blood were the carotid arteries, it might perhaps be possible to intercept a considerable part of it so impelled, and thus remove these symptoms which were the supposed effect of that inordinate influx. With this view, I compressed with my thumb one or both carotids, and uniformly found all the symptoms removed by that process. Those circumstances of rapidity or intensity of thought, which constituted delirium, immediately ceased, and gave place to other trains of a healthy kind; head-ach and vertigo were removed, and a stop was put to convulsions, which the united strength of three or four attendants had before been insufficient to counteract.

That this extraordinary effect was not that of mere pressure, operating as a sort of counteracting stimulus, was evident: for the salutary effect was exactly proportioned to the actual pressure of the carotid itself, and did not take place at all, if, in consequence of a wrong direction either to the right or left, the carotid escaped the effects of the operation.

This view of the order of phœnomena was, in reality, very conformable to the known laws of the animal œconomy. It is admitted, that a certain momentum of the circulating blood in in the brain is necessary to the due performance of the functions of that organ. Reduce the momentum, and you not only impair those functions, but, if the reduction go to a certain degree, you bring on syncope, in which they are for a time suspended. On the other hand, in nervous affections, the sensibility and other functions of the brain are unduly increased; and what can be more natural than to attribute this effect to the contrary cause, or excessive momentum in the vessels of the brain? If, however, this analogical reasoning has any force in ascertaining the principle, I must acknowledge that it did not occur to me till twenty years afterwards, when a great number of direct experiments had appeared to me clearly to demonstrate the fact.

From various cases of this kind, I beg leave to select one which occurred to me in the month of January, 1805.

Mrs. T. aged 51, two years and a half beyond a certain critical period of female life, a widow, mother of two children, thin and of a middle size, had been habitually free from gout, rheumatism, hæmorrhoids, eruptions, and all other disorders, except those usually called nervous, and occasional colds, one of which, about two years and a half before, had been accompanied with considerable cough, and had still left some shortness of breathing, affecting her only when she used strong muscular exertion, as in walking up stairs, or up hill.

In February 1803, after sitting for a considerable time in a room without a fire, in very severe weather, she was so much chilled as to feel, according to her own expression, "as if her blood within was cold." In order to warm herself, she walked briskly for a considerable time about the house, but ineffectually. The coldness continued for several hours, during which she was seized with a numbness or sleepiness of her left side, together with a momentary deafness, but no privation or hebetude of the other senses, or pain or giddiness of the head. After the deafness had subsided, she became preternaturally sensible to sound in the ear of the affected side, and felt a sort of rushing or tingling in the fingers of the left hand, which led her to conclude that "the blood went too forcibly there."

Though the coldness went off, what she called numbness still continued, but without the least diminution of the power of motion in the side affected. In about six weeks, the numbness extended itself to the right side.

Among various ineffectual remedies for these complaints, blisters were applied to the back, and the inside of the left arm above the elbow. The former drew well. The latter inflamed without discharging: so that a poultice of bread and milk milk was put on the blistered part. After this period, the muscles of the humerus began to feel as if contracted and stiff; and these sensations gradually spread themselves to the neck and head, and all across the body, so as to make it uncomfortable for her to lie on either side, though there was no inability of motion.

She now began to be affected with violent occasional flushings of her face and head, which occurred even while her feet and legs were cold, together with a rushing noise in the back of the head, especially in hot weather, or from any of those causes which usually produce the feelings of heat.

It is difficult to give intelligible names to sensations of a new and uncommon kind. That, which this lady denominated numbness, diminished neither the motion nor the sensibility of the parts affected. It was more a perception of tightness and constriction, in which the susceptibility of feeling in the parts was in fact increased; and the skin of the extremities was so tender, that the cold air produced a sense of uneasiness, the finest flannel or worsted felt disagreeably coarse, and the attempts to stick a pin with her fingers caused intolerable pain.

In the month of September 1803, not long after the application of the blisters, she experienced in certain parts of the left arm and thigh, that sensation of twitching which is vulgarly called the "life blood," and which soon extended itself to the right side. Shortly afterwards, she began to perceive an actual vibration or starting up of certain portions of the flexor muscles of the fore-arm, and of the deltoid on the left side; not so, however, as to move the arm or hand.

This disorder had continued with little variation to the period of my first visit. The vibrations constantly existed while the arm was in the common posture, the fore-arm and hand leaning on the lap. If the arm were stretched strongly downward, the vibration of the flexors ceased, but those of the deltoid continued. The arm being strongly extended forwards, all ceased; but returned as soon as the muscles were relaxed. The vibrations were of different degrees of frequency, and at pretty regular intervals, usually about 80 in a minute. They were increased in frequency and force by any thing which agitated or heated the patient, and were always worse after dinner than after breakfast. The pulse in the radial artery was 80 in a minute, and rather hard. That in the carotids was very full and strong; and each carotid appeared to be unusually dilated for about half an inch in length, the adjacent portions above and below being much smaller, and of the natural size. I much regret that I find in my notes of this case, no inquiry whether there was any coincidence coincidence between the systoles of the heart, and the muscular vibrations. The patient's feet were usually cold, and her head and face hot. The feeling in her limbs was much as I have above described, except that the sensibility was somewhat less acute than it had been, and she complained of a tightness all over her head, as if it had been bound with a close night-cap. Her sleep was usually sound on first going to bed, but afterwards, for the most part, interrupted by dreaming. Bowels generally costive: appetite moderate: no flatulency or indigestion: tongue slightly furred, without thirst: urine variable, but generally pale.

The late Mr. George Crook, surgeon, was present while I made these examinations; and when we afterwards conversed together, I remarked to him, that if my theory of the usual cause of spasmodic or nervous affections were well founded, I should probably be able to suppress or restrain these muscular vibrations of the left arm, by compressing the carotid artery on the opposite or right side; while little effect might perhaps be produced, by compressing the carotid of the side affected. The event was exactly conformable to my expectation. Strong pressure on the right carotid uniformly stopped all the vibrations, while that on the left had no apparent influence. I may add that these experiments were afterwards, at my request, repeated on this lady in London by Dr. Baillie, and, as he informed me in a letter, with a similar result.

It is perfectly well known to many of the learned Members of this Society, that irritations of the brain, when of moderate force, usually exhibit their effects on the nerves or muscles of the opposite side of the body; and in the case before us, it is difficult to understand how the suspension of these automatic motions could have been produced by this pressure of the opposite carotid, in any other way than by the interruption of the excessive flow of blood through a vessel morbidly dilated; in consequence of which interruption, the undue irritation of the brain was removed, and the muscular fibres permitted to resume their usual state of rest.

From these and many other similar facts, I am disposed to conclude, that irritation of the brain, from undue impulse of blood, is the common though not the only cause of spasmodic and nervous affections; and I can with the most precise regard to truth add, that a mode of practice conformable to this principle has enabled me, during more than twenty years, to cure a vast number of such maladies, which had resisted the usual means.

